# Fatty Liver in Fish: Metabolic Drivers, Molecular Pathways and Physiological Solutions

**DOI:** 10.3390/ani16020236

**Published:** 2026-01-13

**Authors:** Xiyu Xie, Chaoyang Zhang, Ilham Zulfahmi, Esau Mbokane, Quanquan Cao

**Affiliations:** 1 College of Animal Science and Technology, Sichuan Agricultural University, Chengdu 611130, China; 2 Department of Fisheries Resources Utilization, Faculty of Marine and Fisheries, Syiah Kuala University, Banda Aceh 23111, Indonesia; 3 Marine and Coastal Resources Institute, Faculty of Environmental Management, Prince of Songkla University, Hat Yai 90110, Thailand; 4 Aquaculture Research Unit, School of Agricultural and Environmental Sciences, University of Limpopo, Private Bag X1106, Sovenga 0727, South Africa

**Keywords:** fish, fatty liver, triggering pathway, signal transduction, aquaculture

## Abstract

Fatty liver in fish is characterized by excessive lipid accumulation, driven by factors such as inflammation, oxidative stress, and the overexpression of lipid-related genes. This condition can lead to metabolic dysfunction and reduced disease resistance, resulting in growth disorders and even mortality. Increasing incidence of fatty liver is closely linked to environmental conditions and feeding practices, posing significant challenges to the aquaculture industry. This paper offers a comprehensive overview of hepatic steatosis, with a particular emphasis on fish species. Through a detailed review of various scholarly works, this paper seeks to identify common patterns, emerging trends, and measurable correlations, highlighting the critical importance of understanding this complex relationship. The study of fatty liver is conducted across three dimensions: influencing factors, underlying mechanisms, and potential solutions. Currently, numerous factors contribute to the development of fatty liver, such as feed composition and environmental temperature. On a mechanistic level, the research explores lipid accumulation, inflammation, oxidative stress, and related processes. Furthermore, the paper suggests various solutions and preventive strategies, including considering environmental adaptability during animal migration, employing genetic enhancement techniques, modifying feeding practices, investigating the Nrf2 pathway, and utilizing rapamycin. These findings have significant implications for fisheries management and aquaculture practices, providing valuable insights to enhance sustainability in the industry.

## Simple Summary

This study addresses the growing issue of fatty liver disease in farmed fish, a metabolic disorder primarily driven by nutritional imbalances, particularly diets high in carbohydrates and lipids, and exacerbated by intensive aquaculture practices. The aim was to synthesize the current understanding of its pathological drivers, including disrupted lipid metabolism and oxidative stress, and the underlying molecular pathways involved. The analysis concludes that the condition severely impacts fish health, growth, and survival. Key solutions identified include dietary interventions, such as optimizing feed composition with functional ingredients, and improved farming management, which are crucial for preventing this disorder and ensuring sustainable aquaculture.

## 1. Introduction

As the global population continues to grow rapidly and economies expand, meeting the demand for animal products has become an unavoidable challenge. Fish, serving as a paramount reservoir of premium protein, indispensable fatty acids, and vitamins, is instrumental in augmenting human nutritional standards. This has catalyzed a persistent upsurge in the requirement for aquatic commodities [1,2]. In the future, aquatic products are expected to become the primary source of aquatic food, with the added benefit of indirectly protecting the world’s wild fisheries.

Lipids constitute fundamental components in piscine growth and ontogeny. They fulfil critical physiological roles by furnishing energy, essential fatty acids, and fat-soluble nutrients for fish [3,4]. The optimal growth performance of crucian carp (*Carassius carassius*) can be achieved with appropriate lipid levels, although excessive lipid levels can promote the digestion of nutrients, they may promote hepatic lipid accumulation and predispose fish to liver injury to some extent [5]. Long-chain polyunsaturated fatty acids are important metabolic and immune function regulators for salmon fish and are crucial for disease prevention [6]. The fat content of fish varies greatly, generally ranging from 1% to 10%. Fish like cod and abalone have less than 1% fat, whereas eel, shark, sea bass, and salmon are among the fatty fish with fat content exceeding 10% [7].

Environmental factors such as heavy metal pollution and excessive chemical drugs can induce the occurrence of fatty liver in fish. Heavy metals in aquaculture water can reduce the antioxidant capacity of fish, lead to liver lipid metabolism disorder, and cause excessive fat deposition [8]. Numerous studies have shown that a variety of heavy metals, such as cadmium, mercury, lead, etc., can disrupt the normal function of cells through toxic effects, resulting in metabolic imbalance and fat deposition in tissues, especially in the liver [9]. The pathogenesis of piscine fatty liver is principally attributable to an imbalance in requisite nutrients and a deficit of certain hepatoprotective factors. This leads to a dysregulation of hepatic lipid metabolism, resulting in its deposition, infiltration, and an overall augmentation in hepatic fat content, which can progress to pathological steatosis when accompanied by cellular damage, thereby precipitating the condition [4,10]. Managing these dietary components is crucial to maintaining optimal liver health and overall well-being in farmed fish. When these fats reach the liver and are not transported out in a timely manner, they can accumulate within the liver, leading to metabolic dysfunction [10]. High-lipid dietary regimens in piscine feed can provoke an inordinate accumulation of lipids in the liver, leading to hepatic dysfunction and fatty infiltration, and this state may evolve into fatty liver disease [11,12]. Typically, fatty liver disease is diagnosed when the lipid content in the fish liver exceeds 5% of the liver’s wet weight and is associated with histopathological changes such as hepatocyte ballooning, inflammation, or fibrosis [11].

However, it is crucial to recognize that hepatic lipid accumulation is not inherently pathological. In fact, it represents a fundamental physiological adaptation in fish. This is evident in Nile tilapia (*Oreochromis niloticus*), which can maintain stable hepatic triglyceride levels across a wide range of dietary lipid intake (from 1% to 13%) by activating adaptive metabolic pathways, demonstrating the liver’s central role in lipid homeostasis [13]. Similarly, the Atlantic cod liver naturally stores 50–70% of its total lipid content as triglycerides within hepatocytes, forming a specialized energy reservoir crucial for its physiology [14]. Under natural conditions, many fish species exhibit seasonal cycles of lipid deposition and mobilization in the liver, which are essential for their survival and reproduction. For instance, prior to overwintering, some species can accumulate hepatic lipids exceeding 15% of liver weight, serving as a critical energy reserve to sustain metabolism during periods of low temperature and food scarcity [15,16]. Similarly, during the reproductive season, broodstock often develop significant hepatic steatosis, as the liver acts as a primary site for synthesizing and storing lipids that are subsequently transported to the developing gonads to support vitellogenesis and embryogenesis [17,18]. These evolutionarily conserved strategies highlight the liver’s central role in energy management and life history strategies in fish. Therefore, the pathological condition of “fatty liver disease” in aquaculture must be understood as a dysregulation of this essential physiological process, as demonstrated by studies where exposure to environmental pollutants (e.g., PFAS) or specific PPAR agonists can disrupt lipid homeostasis, leading to altered lipid transport, composition, and potential steatotic responses in fish livers [14]. This condition is typically triggered by persistent, non-physiological challenges such as chronic high-fat diets, environmental stressors, or sedentarity, which disrupt the homeostatic balance between lipid storage and mobilization.

Therefore, it is crucial to accurately distinguish between physiological fat storage and pathological fatty liver disease. Physiological accumulation is a highly regulated, reversible process synchronized with energy demands and reproductive cycles, typically not accompanied by severe oxidative stress, inflammation, or cellular damage. In contrast, pathological fatty liver disease, particularly in intensive aquaculture settings, results from the breakdown of metabolic regulatory networks. Key markers include: persistent abnormal upregulation of lipid synthesis genes (e.g., SREBP-1c, FASN), inhibition of fatty acid β-oxidation (e.g., reduced CPT1a expression), impaired antioxidant defense systems, and abnormal activation of pro-inflammatory factors (e.g., TNF-α, IL-1β) and cell death pathways [19,20,21,22]. This metabolic imbalance is often triggered by sustained non-physiological stressors such as prolonged high-fat/high-fructose diets, environmental stressors (hypoxia, temperature extremes), or restricted activity. Future research should focus on identifying critical metabolic nodes and early biomarkers that mark the transition from adaptive storage to pathological states, which holds significant implications for early warning and precision intervention in aquaculture practices.

The aquaculture industry faces numerous challenges, among which fatty liver injury stands out as a common liver disease in farmed fish worldwide, particularly severe in China. This fat accumulation can be triglycerides, fatty acids, phospholipids, or cholesterol esters. Fatty liver can induce metabolic disorders, attenuate disease resistance, and even cause mortality in fish [23]. Despite its widespread prevalence, the precise pathogenic mechanisms underlying fatty liver disease remain incompletely understood. However, persistent and dedicated research efforts by numerous scholars have yielded valuable insights into its induction mechanisms. Fatty liver disease is influenced by a variety of factors, including inflammation, oxidative stress, and other contributing elements, which will be discussed in detail in the following sections. A deeper understanding of these factors not only enhances preventive strategies for fatty liver but also provides potential directions for its treatment.

Reducing the intake of high-fat or high-fructose diets and considering environmental adaptability during animal migration can help lower the risk of developing fatty liver. Moreover, advancements in genetic research have opened new avenues for treating fatty liver. These include inhibiting mechanistic target of rapamycin (mTOR) signaling through rapamycin to reduce fat accumulation, reversing oxidative stress, or utilizing genetic modification to mitigate the onset of fatty liver. Rapamycin acts as an upstream regulator of mTOR, inhibiting its expression. It stabilizes the inhibitors of the mTOR signaling pathway and downregulates mTOR activity. Investigations have confirmed that rapamycin-mediated inhibition of mTOR diminishes the phosphorylation of proteins within the mTOR signaling pathway, induces cell cycle arrest at the G0/G1 phase, and markedly reinstates the differentiation of normal hematopoietic stem cells (HSCs) while exhausting leukemic stem cells. These findings highlight the potential of targeted therapeutic approaches in addressing fatty liver disease and its underlying mechanisms [24].

**Figure 1:**
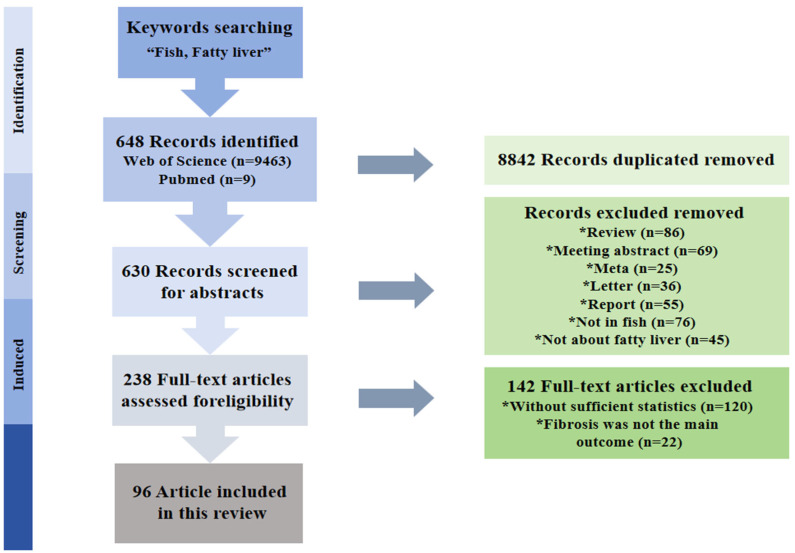
Flowchart of Literature Screening and Study Inclusion for the Review on Fatty Liver Disease in Fish (PRISMA Diagram). This flowchart, developed in accordance with the Preferred Reporting Items for Systematic Reviews and Meta-Analyses (PRISMA) guidelines, illustrates the systematic process of identifying, screening, and selecting relevant studies for this review on fatty liver disease in fish. 1. Search and Initial Identification: A comprehensive literature search was conducted using the key terms “Fish, Fatty liver” across two major databases: Web of Science (*n* = 9463) and PubMed (*n* = 9). In total, 9472 records were identified. After removing 8842 duplicates, 630 unique records remained for title and abstract screening. 2. Abstract Screening and Full-Text Assessment: During the title and abstract screening phase, 392 records were excluded based on predefined exclusion criteria, including: review articles (*n* = 86), meeting abstracts (*n* = 69), meta-analyses (*n* = 25), letters or commentaries (*n* = 36), reports (*n* = 55), studies not conducted in fish (*n* = 76), and studies not focused on fatty liver (*n* = 45). This resulted in 238 articles that proceeded to full-text evaluation. 3. Full-Text Screening and Final Inclusion: Following full-text review, an additional 142 articles were excluded due to insufficient statistical data (*n* = 120) or because fibrosis was not a primary outcome (*n* = 22). Ultimately, 96 articles met all eligibility criteria and were included in this systematic review for further analysis and synthesis. Notes: Exclusion categories and corresponding numbers are specified at each screening stage, ensuring transparency and reproducibility of the selection process. This structured approach guaranteed the relevance and quality of the included studies, providing a methodologically sound foundation for the subsequent review. * is just a list label.

**Figure 2:**
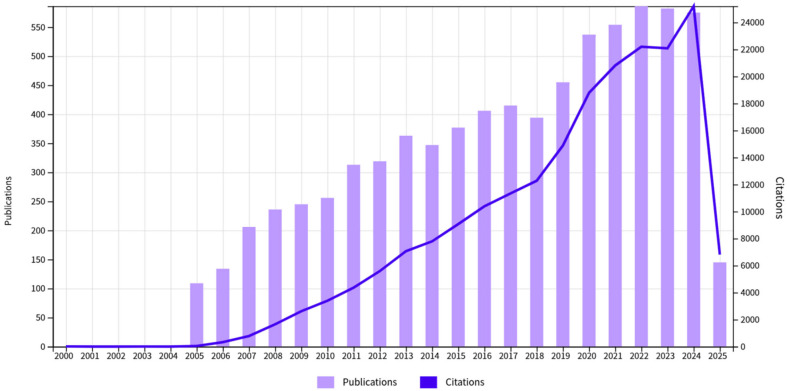
The changes in the number of citations and publishers related to fatty liver over the years, indicating that fatty liver is garnering increasing attention from the public and research fields (21 April 2025).

The number of references and publishers related to fatty liver is increasing year by year and its status in public discourse is also increasing, which brings both opportunities and challenges (Figure 1 and Figure 2). Moving forward, we explore the future prospects of research on fish fatty liver, highlighting the need for more comprehensive studies to uncover the regulatory mechanisms of lipids across various fish species and environmental conditions. Lastly, this article will focus on outlining potential directions for developing effective solutions to address fatty liver disease.

## 2. The Factors and Mechanism of Triggering Fatty Liver

### 2.1. Factors

#### 2.1.1. Feed Composition

Lipids, serving as a non-protein energy substitute, are crucial for aquatic animals, providing essential fatty acids and other lipids [25]. Moreover, dietary lipid supplementation can mitigate the entry of nitrogen-containing waste into aquatic environments [23]. However, excessive fat intake can hinder growth and disrupt lipid metabolism, a conclusion that has been confirmed in tilapia, Japanese seabass (*Lateolabrax japonicus*), blunt snout bream (*Megalobrama amblycephala*). [26,27,28,29,30]. In recent years, scientific inquiry has increasingly centered on high-fat diet (HFD)-elicited hepatic injury in fish.

Scholarly works examining the impact of high-fat diets on lipid deposition in fish disclose that HFD not only instigates hepatic fat accumulation but also induces oxidative stress and chronic inflammation [26,27,28,29]. High-fat diets disrupt lipid metabolism in tilapia liver, elevate liver lipid peroxidation, and compromise liver immune status [25,31]. High-fat diets impair the antioxidant and immune functions of intestinal cells in freshwater drum (*Aplodinotus grunniens*), promote inflammation, cellular apoptosis, and autophagy, and reduce intestinal microbial diversity, thereby disrupting the ecological balance of the gut microbiota [32]. A HFD frequently displays excessive hepatic lipid deposition and heightened activities of plasma alanine aminotransferase (GPT) and aspartate aminotransferase (GOT), which are classical biomarkers of hepatic injury [33]. Ergo, elevated dietary lipid content markedly amplifies the risk of developing fatty liver by promoting primary lipid accumulation and creating a pro-inflammatory and pro-oxidant environment conducive to pathological progression (Table 1). However, recent research suggests an initial adaptive phase. In Nile tilapia, short-term (4-week) high-fat diet (HFD) intake did not cause liver injury but triggered a protective response characterized by hepatic cholesterol ester accumulation and enhanced cholesterol–bile acid flux, potentially through stimulated peroxisomal fatty acid β-oxidation [34].

Nonalcoholic fatty liver disease (NAFLD) involves the accumulation of surplus fat within hepatocytes, encompassing a spectrum of disorders indicative of fibrosis severity, with potential progression to hepatocellular carcinoma. In rodent models, fructose intake correlates with augmented lipogenesis, diminished fatty acid oxidation, periportal fibrosis, endoplasmic reticulum (ER) stress, and compromised insulin signaling [35,36]. Fructose therapy can induce hepatic steatosis and activate pathways associated with Nonalcoholic steatohepatitis (NASH). Studies by Valerie Sapp et al. reveal that fructose treatment induces liver lipid accumulation, inflammation, and oxidative stress in zebrafish (*Danio rerio*) fry, with Torc1 activation being crucial for liver lipid accumulation in NAFLD models and patients [37], making fructose a significant inducer of fatty liver in fish.

Fish oil, abundant in highly unsaturated fatty acids (HUFAs) like eicosapentaenoic acid (EPA) and docosahexaenoic acid (DHA), represents an optimal lipid source for piscine diets [38,39,40]. Supplementing tilapia diets with fish oil has been shown to significantly enhance their healthy fat content [41]. Recent research on Nile tilapia further demonstrates that HFD feeding induces time-dependent dynamic alterations in flesh nutrient composition, including reduced phospholipids and n-3 PUFAs, which may reflect systemic metabolic shifts associated with hepatic steatosis [42]. In recent years, citrate has gained attention for its role in regulating nutrient metabolism in fish. As a key intermediate in the tricarboxylic acid cycle, citrate is involved in cytoplasmic acetyl-CoA production, influencing both lipid synthesis and energy metabolism [43]. A study on Nile tilapia. reported that dietary supplementation with 4% sodium citrate significantly increased whole-body crude protein, serum triglycerides, and hepatic glycogen levels, while also promoting protein deposition in muscle via activation of the mTOR signaling pathway [20]. However, this treatment also induced hyperglycemia, insulin resistance, and activation of inflammatory signaling pathways in the liver, revealing a double-edged effect of sodium citrate in promoting nutrient deposition at the potential cost of metabolic dysregulation [20].

**Table 1:** The effect of feed composition on fat accumulation in fish.

Feed Composition	Impact	Fish	References
Dietary lipid	Excessive fat intake can hinder growth and disrupt lipid metabolism, inducing liver damage in fish.	Tilapia	Qiang et al. (2017); Tao et al. (2018) [25,31]
Fructose	Fructose treatment induces hepatic lipid accumulation, inflammation, and oxidative stress.	Zebrafish fry	Sapp et al. (2014) [37]
Fish oil	Fish oil is an ideal lipid source for a fish-based diet.	Tilapia	Kohen et al. (2002); Radovanović et al. (2010); Stoneham et al. (2018) [38,39,41]

#### 2.1.2. High Feeding Rate

The makeup and frequency of feed deliveries exert a significant influence on the progression of fatty liver. High feeding rates not only promote lipid accumulation but also trigger oxidative stress and other adverse effects. In largemouth bronze gudgeon (*Coreius guichenoti*), high feeding rates markedly elevated the levels of various lipid indicators, such as triglycerides, in the liver. This led to enhanced liver fat synthesis, reduced fat breakdown, and ultimately, the accumulation of liver fat. It also triggered oxidative stress and inflammatory responses, exacerbating liver damage [44]. In experiments with elevated feeding rates, levels of various liver markers such as Total Bile Acids (TBA), Total Cholesterol (TC), Non-Esterified Fatty Acids (NEFAs), Alanine Aminotransferase (ALT), Aspartate Aminotransferase (AST), Alkaline Phosphatase (AKP), and total bile significantly increased, while Catalase (CAT) and Superoxide Dismutase (SOD) activity decreased. Concentrations of immune markers such as Immunoglobulin G (IgG), Immunoglobulin M (IgM), and Lysozyme (LYS) also escalated, alongside inflammatory cytokines Tumor Necrosis Factor-alpha (TNF-α) and Interleukin-1 beta (IL-1β), a phenomenon confirmed in largemouth bronze gudgeon [44]. Red hybrid tilapia subjected to a feeding rate exceeding 3% developed a fatty liver phenotype due to enhanced lipid accumulation, which further intensified oxidative stress and inflammation [45]. Furthermore, a high feeding frequency not only increases breeding costs but also adversely affects fish growth, damages internal organs, impairs feed digestion and absorption, and contributes to water pollution. The feeding level plays a crucial role in influencing fish growth, body composition, and feed efficiency. Therefore, it is essential to develop optimal feeding strategies to minimize individual growth disparities and enhance the economic benefits of aquaculture.

#### 2.1.3. Environment

Stress, including cold stress, triggers adaptive behavioral and physiological responses in common carp (*Cyprinus carpio*) to cope with changing aquatic environments [46]. Studies on cold stress have underscored enhancements in antioxidative capacity, glucose metabolism, and fatty acid metabolism during cold acclimatization [47,48,49,50]. Differential expression of genes related to glucose and lipid metabolism and mTOR signaling pathways is observed during cold stress, indicating liver damage and fatty liver induction [51].

Hypoxia, defined by an inadequate supply of oxygen in the biological environment, is a crucial environmental factor that significantly impacts fish survival, growth, behavior, reproduction. Low oxygen stress induces oxidative damage, inhibits growth, and alters serum biochemical and immune indicators, impacting material metabolism and immune function [52]. Oxidative stress is a key driver of hepatic steatosis and obesity, which leads to lipid accumulation and cellular damage [53].

Another significant factor contributing to abnormal fat accumulation is Survival habits. Research has demonstrated that wild fish generally possess lower fat content compared to their farmed counterparts. For instance, wild Atlantic salmon (*Salmo salar*) exhibit body fat percentages below 15%, whereas intensively farmed conspecifics often exceed 20%, with hepatic steatosis being a common pathological finding [54]. This disparity is not merely nutritional but rooted in evolutionary metabolic adaptations. The imperative for wild fish to undertake extensive reproductive migrations (e.g., salmonid anadromy) and evade predators necessitates sustained high-energy expenditure, primarily fueled by lipid catabolism. In contrast, the controlled aquaculture environment is characterized by hydrological stability and physical confinement, which systematically eliminates the ecological drivers and spatial possibilities for such natural migratory behaviors [55].

### 2.2. Mechanism

#### 2.2.1. Abnormal Fat Accumulation

Fish exhibit seasonal hepatic lipid accumulation in natural environments. For instance, prior to winter, fish accumulate over 15% of lipid as energy reserves for cold periods. During the breeding season, parent fish store hepatic fat to provide lipid precursors for gonadal development. These adaptive traits have evolved through species adaptation.

**Figure 3:**
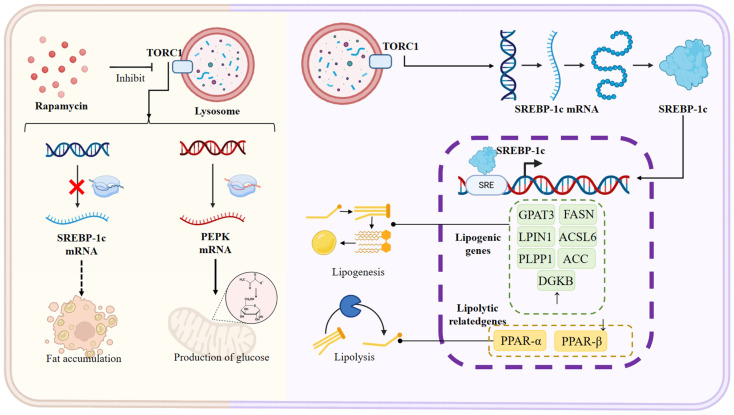
The Central Regulatory Network of the mTORC1 Signaling Pathway in Hepatic Lipid Synthesis and Catabolism. This schematic diagram systematically illustrates the key role and molecular mechanisms of the mammalian target of rapamycin complex 1 (mTORC1) in regulating hepatic lipid homeostasis. The left portion depicts the metabolic programming under mTORC1 activation: activated mTORC1 promotes lipid accumulation through a dual mechanism — on one hand, it significantly upregulates the transcription and expression of sterol regulatory element-binding protein 1c (SREBP-1c), thereby activating its downstream network of lipogenic genes (including ACC, ACSS2, SCD1, GPAT3, FASN, LPIN1, etc.), which drives de novo synthesis of fatty acids and triglycerides; on the other hand, it simultaneously inhibits the autophagy–lysosome pathway and lipolysis, reducing lipid breakdown. The right portion presents the metabolic reprogramming following mTORC1 inhibition: treatment with rapamycin or inhibition of mTORC1 activity relieves the suppression of the autophagy–lysosome pathway, enhancing lipid degradation; concurrently, it downregulates SREBP-1c and its target gene expression while upregulating lipolysis-related genes (such as PPAR-α and PPAR-β), synergistically promoting lipid mobilization and clearance. The diagram also specifically highlights mTORC1’s regulation of the gluconeogenic key enzyme PEPCK, revealing its pivotal role in the cross-regulation of glucose and lipid metabolism. Solid arrows indicate direct or experimentally verified regulatory relationships, while dashed arrows represent indirect or potential regulatory pathways.

Abnormal fat accumulation is a key initial step in fatty liver development, driven by diet and environment. It mainly involves two mechanisms: increased lipid production and decreased fat breakdown. Previous studies have demonstrated an abnormal increase of adipogenic genes, such as cidc and lipin1, development of hepatic steatosis. Concurrently, elevated levels of adipogenic transcription factors, including srebf1, further indicate increased lipid production during this phase. Specifically, the master regulator SREBP-1c activates downstream lipidogenic genes (Figure 3), creating a cascade that drives hepatic adipogenesis. Additionally, mTOR (mechanistic target of rapamycin) has surfaced as a key governor of cell growth and metabolism, encompassing adipogenesis, protein translation, and autophagy [56]. Recent studies intimate that mTOR signaling may represent a potential pathway in nonalcoholic steatohepatitis (NASH), with Torc1 activation correlated with increased hepatic lipogenesis in rodent models [57]. As illustrated in the diagram, mTOR’s downstream effects are mediated through SREBP-1c, which activates the entire adipogenesis program when not inhibited (Figure 3). Genetic ablation of S6K1, a downstream effector of Torc1, has been shown to reduce hepatic lipid accumulation in obese mice. Notably, Torc1 serves as a key integrative factor assimilating data pertinent to cellular energy and nutritional status, growth factor signaling, and hypoxia. Therefore, this pathway may also intersect with others, such as oxidative stress. Interestingly, studies have demonstrated that increased Torc1 activity can paradoxically reduce liver steatosis. Liver-specific knockout of Tsc1, a negative regulatory factor of Torc1, in mice increases Torc1 activity, thereby preventing age- and diet-induced hepatic steatosis [58,59].

However, the role of mTOR in hepatic lipid metabolism is highly context-dependent and often paradoxical, presenting a significant challenge for therapeutic targeting. On one hand, sustained nutrient-activated mTORC1 promotes lipogenesis by stabilizing and activating SREBP-1c [60], a mechanism implicated in diet-induced fatty liver. Consistent with this, the mTOR inhibitor rapamycin can ameliorate hepatic steatosis in some models [37,61]. On the other hand, conflicting evidence shows that constitutive activation of mTORC1 (e.g., via liver-specific Tsc1 knockout) enhances lipid oxidation and suppresses diet-induced steatosis [58,59]. This paradox may be explained by the pleiotropic functions of mTOR and the differential outcomes of acute pharmacological inhibition versus chronic genetic activation. Chronic mTORC1 activation might trigger adaptive feedback mechanisms, such as upregulating mitochondrial biogenesis and β-oxidation to cope with sustained anabolic pressure, whereas acute inhibition by rapamycin primarily blocks the pro-lipogenic arm. Furthermore, the metabolic effects of rapamycin can be counterproductive, as it may concurrently impair glucose homeostasis and induce insulin resistance, as seen in turbot where it disrupted glycolysis [61]. These contradictory findings highlight that mTOR functions as a central hub within a complex signaling network. Its effect on liver fat is not linear but depends on the duration, intensity, and subcellular localization of its activity, as well as interactions with other pathways like AMPK and autophagy. Simplistic “mTOR activation = bad” or “mTOR inhibition = good” paradigms are inadequate. Future research needs to delineate the specific downstream effectors and temporal patterns of mTOR signaling that drive pathological versus protective metabolic outcomes in fish liver.

The metabolic pathway of citrate is also closely linked to lipid accumulation. In Nile tilapia, 4% sodium citrate supplementation significantly elevated hepatic acetyl-CoA and triglyceride contents. However, this was accompanied by the downregulation of key lipogenic genes (such as srebp1, fasn, and acly) and a significant reduction in the expression of CPT1a, a critical enzyme for mitochondrial fatty acid β-oxidation [20]. This suggests that in this model, citrate-induced hepatic lipid accumulation primarily stems from the inhibition of lipid catabolism rather than the enhancement of de novo lipogenesis [20]. Furthermore, the sodium citrate treatment led to increased phosphorylation of p65, indicating a potential activation of the NF-κB pathway and a mild inflammatory response [20]. This dual role of mTOR signaling explains why rapamycin (an mTOR inhibitor) can block SREBP-1c mRNA and lipid accumulation (Figure 3), while paradoxically failing to inhibit gluconeogenic genes like PEPCK, allowing glucose production to continue unabated during treatment. Notably, Torc1, a central regulatory factor that integrates cellular energy and nutrient status, growth factor signaling, and hypoxia responses, may also impinge upon oxidative stress pathways. Intriguingly, while enhanced Torc1 activity can alleviate liver steatosis under certain conditions, its inhibition may promote fat accumulation through feedback mechanisms, highlighting the intricate interplay of regulatory pathways in the development of fatty liver disease.

Hepatic transcriptome sequencing has recognized PPAR (peroxisome proliferator-activated receptor) as intimately associated with lipid metabolism and the genesis of fatty liver. Through transcriptomic analysis of liver tissues from rainbow trout (*Oncorhynchus mykiss*) with high and low body fat content, 1694 differentially expressed transcripts were identified, including genes pertinent to lipid metabolism such as PPAR-α and PPAR-β, indicating that the hepatic lipid metabolic process may be correlated with disparities in body fat content [62]. These findings complement the diagram’s representation of fat production genes being continuously enhanced during fatty liver development. Analysis of lipid-metabolism-related differentially expressed genes (DEGs) indicates significant upregulation of lipid synthesis-associated genes such as GPAT3, LPIN1, PLPP1, FASN, ACC1, DGKB, and ACSL6 during fatty liver formation. These findings suggest that increased fat synthesis coupled with decreased fat breakdown leads to liver fat accumulation, ultimately triggering oxidative stress and inflammatory reactions, exacerbating liver damage, and the results have been confirmed in largemouth bronze gudgeon [44]. This aligns with the diagram’s depiction of sustained fatty acid synthesis and glucose production pathways operating in parallel during disease progression (Figure 3). Additionally, mTOR (mechanistic target of rapamycin) has surfaced as a key governor of cell growth and metabolism, encompassing adipogenesis, protein translation, and autophagy. Recent studies intimate that mTOR signaling may represent a potential pathway in nonalcoholic steatohepatitis (NASH), with Torc1 activation correlated with increased hepatic lipogenesis in rodent models [57].

#### 2.2.2. Oxidative Stress

The oxidative stress response affords valuable understanding into the malfunction or partial failure of cellular defense mechanisms against Reactive Oxygen Species (ROS) [63]. Reactive Oxygen Species (ROS) are crucial for immune modulation, upholding redox equilibrium, and initiating cellular signaling pathways [64,65,66]. However, disproportionate ROS generation can impair cellular lipids, proteins, nucleic acids, membranes, and organelles, leading to the initiation of apoptotic pathways and contributing to cellular degradation [63,67,68,69] Recent studies have shown that antibiotic exposure, such as oxytetracycline (OTC), can exacerbate oxidative stress by impairing mitochondrial function and antioxidant enzyme systems, further contributing to hepatic damage in largemouth bass (*Micropterus salmoides*) [70,71].

**Figure 4:**
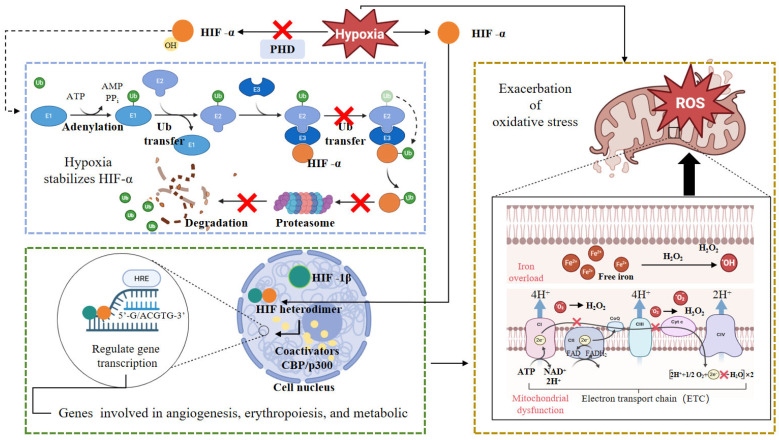
Integrated Regulation of HIF-α Stability and Iron-Mediated Oxidative Stress in Hypoxic Adaptation. This schematic illustrates the molecular control of HIF-α under varying oxygen conditions and its interface with iron-induced oxidative stress. Under normoxia, prolyl hydroxylase domain enzymes (PHDs) hydroxylate HIF-α, leading to its ubiquitination and proteasomal degradation. Hypoxia stabilizes HIF-α by inhibiting PHD activity, allowing HIF-α to translocate to the nucleus, dimerize with HIF-1β, recruit transcriptional coactivators (CBP/p300), and induce the expression of genes involved in angiogenesis, erythropoiesis, and metabolic adaptation. Concurrently, iron overload exacerbates oxidative stress by promoting the Fenton reaction, in which free iron catalyzes the conversion of H_2_O_2_ into highly reactive hydroxyl radicals. This oxidative burden contributes to mitochondrial dysfunction, particularly affecting the electron transport chain (ETC). The diagram suggests a potential crosstalk between hypoxia signaling and iron-driven oxidative pathways, highlighting their combined role in cellular stress responses and metabolic regulation. Solid arrows denote direct regulatory steps, while dashed arrows indicate indirect or modulatory interactions.

As illustrated in the diagram, hypoxia serves as a key trigger for ROS production, with factors like iron and mitochondrial dysfunction exacerbating oxidative stress. This creates a vicious cycle where hypoxia-induced ROS further destabilizes cellular homeostasis (Figure 4). Exposure to oxygen-deficient environments has been associated with elevated levels of reactive oxygen species (ROS), inducing oxidative stress with injurious effects on numerous cell types. Intracellular iron may aggravate oxidative stress, leading to ferroptosis. Ferroptosis is an iron-dependent programmed cell death triggered by lipid peroxidation accumulation, and its core mechanism is the failure of the antioxidant defense system of glutathione peroxidase 4 (GPX4). Lipid peroxidation ensuing from redox imbalance is a fundamental mechanism underlying lipid toxicity in the liver [72,73]. Under normoxic conditions, HIF-α undergoes hydroxylation, leading to its degradation. However, hypoxia stabilizes HIF-α, allowing it to form heterodimers with HIF-1β that translocate to the nucleus (Figure 4). Hypoxia-inducible factor (HIF-1α) is involved in oxidative stress, serving as a principal regulator of cellular adaptation to hypoxic stress [74,75,76]. While elevated HIF-1α levels facilitate cellular acclimatization to hypoxia, excessive oxidative stress can impair HIF-1α function, leading to deleterious effects on cellular physiology. As illustrated, the HIF heterodimer binds to hypoxia-responsive elements (HREs) in the genome, recruiting co-activators like CBP/p300 to modulate transcription of target genes involved in angiogenesis, erythropoiesis, and metabolic adaptation (Figure 4). Additionally, oxidative stress disrupts mitochondrial quality control, adversely impacting both mitochondrial function and structure under conditions of oxidative stress [77].

The interconnection between oxidative stress and a high-fat diet (HFD) is substantial, with fat accumulation increasing vulnerability to oxidative stress and weakening the antioxidant defence system [78,79,80]. Research found that HFD feeding significantly impaired the growth performance of Orange-spotted grouper (*Epinephelus coioides*), leading to excessive deposition of liver and abdominal fat, inhibiting the activity of antioxidant enzymes, and increasing markers of lipid and protein peroxidation [81]. Enhanced lipid peroxidation, protein oxidation, and DNA damage exacerbate HFD-induced liver damage [82]. Moreover, oxidative stress suppresses the antioxidant defense system, including key regulatory factors like Nrf2 (NF-κE2-related factor 2, a core transcription factor of Cap ‘n’ Collar transcription factor family belonging to leucine zipper family, is involved in cellular resistance to oxidative stress), contributing to mitochondrial dysfunction and liver injury in HFD-fed fish [19,56,83].

Regarding the induction of oxidative stress, it is characterized by the overproduction of free radicals, such as reactive oxygen species (ROS) and reactive nitrogen species (RNS). When oxidation surpasses the body’s capacity for removal, it leads to cellular and tissue damage [38]. Oxidative stress performs a critical function in the progression from simple steatosis to nonalcoholic steatohepatitis (NASH), a liver affliction [21,84]. It is regarded as a major contributor to liver disease advancement, causing mitochondrial dysfunction, cellular apoptosis, and inflammation [78,79]. Prior research has indicated a connection between endoplasmic reticulum (ER) stress and hepatic steatosis, suggesting a close relationship between ER stress and oxidative stress [85,86]. Additionally, oxidative stress has been shown to directly induce hepatic steatosis, as corroborated by the accumulation of lipids in the liver of zebrafish larvae exposed to the oxidative stress inducer valinomycin. This highlights that oxidative stress is not only a consequence but also a driver of lipid dysregulation.

Recent studies have evidenced that specific amino acids can markedly enhance hepatic antioxidant capacity and alleviate endoplasmic reticulum (ER) stress and autophagy under pathological conditions. Dietary valine supplementation activated the Nrf2/Keap1 signaling pathway, upregulated antioxidant enzyme activities (SOD, CAT, GST, GPx, GR), and reduced oxidative stress markers (ROS, MDA, PC) in the liver of Largemouth bass infected with *Aeromonas veronii*. Additionally, valine attenuated ER stress-induced apoptosis and suppressed excessive autophagy, highlighting the potential of amino acid supplementation in managing oxidative stress and metabolic dysfunction in fish liver [2].

#### 2.2.3. Inflammation

**Figure 5:**
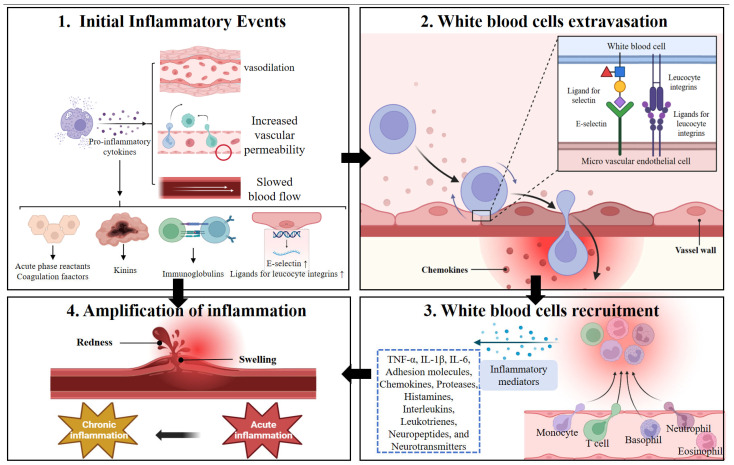
The Inflammatory Cascade in Four Stages. This diagram outlines the sequential stages of the inflammatory response, from initiation to outcome. 1. Initial Events: Pro-inflammatory cytokines trigger vascular changes (vasodilation, increased permeability) and upregulation of adhesion molecules (e.g., E-selectin), preparing the tissue for leukocyte recruitment. 2. Leukocyte Extravasation: Circulating white blood cells roll, adhere, and transmigrate across the activated endothelium into the tissue, guided by chemokines. 3. Recruitment and Mediator Release: Infiltrating leukocytes (neutrophils, monocytes, etc.) release cytokines, chemokines, proteases, and other mediators, amplifying the inflammatory signal. 4. Amplification and Outcome: A self-sustaining cycle of inflammation results in redness, swelling, and tissue damage. The response may resolve, persist acutely, or progress to chronic inflammation. Solid arrows indicate direct progression.

Inflammation is the immediate local immune response of the body to injury or harmful stimuli, which has been confirmed in zebrafish [87] Typical inflammatory symptoms include increased vascular permeability, swelling, heightened blood flow, redness, and nerve fiber sensitization leading to pain [88,89,90]. As illustrated, these cardinal signs of inflammation stem from a cascade initiated by various triggers that enhance blood flow, induce vasodilation, and increase vascular permeability—processes mediated by microvascular endothelial cells (Figure 5). This reaction can occur within minutes to hours and may last for days or weeks. The types of cells and mediators involved in inflammation vary depending on factors such as timing, triggers, anatomical location, and severity of the inflammation [91]. The diagram shows how inflammatory mediators stimulate the production of key immune components (immunoglobulins, kinins, acute phase reactants, and coagulation factors) while prompting endothelial cells to express E-selectin and increase ligands for leukocyte integrins—critical steps for the recruitment of white blood cells to the inflammatory locus (Figure 5). In the attempt to reinstate physiological homeostasis, inflammation may persist and become chronic [92]. For example, tilapia fed a high-fat diet for 60 days displayed significantly higher plasma levels of TNF-α and IL-1β relative to the control group [19]. This chronic low-grade inflammation is a hallmark of the transition from simple steatosis to steatohepatitis.

During inflammation, circulating immune cells, including monocytes, granulocytes, and T-cells, are recruited to the inflammatory site via the secretion of inflammatory mediators, marking an essential step in commencing the inflammatory response [93,94]. This cellular recruitment process is visually depicted in the diagram, illustrating how chemokines mediate leukocyte migration to amplify the inflammatory response at the affected site (Figure 5). Various preformed inflammatory mediators, such as TNF-α, IL-1β, IL-6, adhesion molecules, chemokines, proteases, histamines, interleukins, leukotrienes, neuropeptides, and neurotransmitters, are released, contributing to the inflammatory process. This is consistent with the findings of Xiao Juan Zhang et al. [21,91,95,96].

Chronic inflammation is frequently observed in diverse animal models fed a high-fat diet (HFD), including murine and piscine models, which can disrupt hepatic lipid metabolism and worsen liver injury [97,98,99]. This inflammatory milieu is also a key driver of liver fibrosis, promoting the activation of HSCs and collagen deposition [12]. Recent studies have further demonstrated that dietary supplementation with glycyrrhetinic acid (GA) can attenuate HFD-induced intestinal inflammation and pyroptosis in largemouth bass, highlighting its potential as an anti-inflammatory agent in aquaculture [83]. The diagram’s representation of inflammation amplification helps explain how persistent triggers (like HFD) can transform acute inflammatory responses into chronic conditions through continuous mediator production and leukocyte recruitment (Figure 5). Multiple signaling pathways, such as Toll-like receptor (TLR), JNK, and NFkB pathways, regulate the development of chronic inflammation in fatty liver injury [100]. After 90 days of HFD feeding, NF-κB levels (including TNF-α and IL-1β) and mRNA expression of IL-8, IL-6, and IL-10 were elevated in tilapia. Conversely, IL-6 and interleukin-1 levels were significantly reduced after 60 days of HFD [19]. Conversely, levels of IL-6 and interleukin-1 were significantly reduced after 60 days of HFD [19]. Additionally, HFD activates the TLRs (TLR1 and TLR2)-MyD88 pathway, promoting inflammation and liver damage in tilapia. This activation may be associated with elevated free fatty acids (FFAs) and reactive oxygen species (ROS) [101]. Furthermore, recent studies have shown that dietary vitamin D_3_ supplementation can ameliorate hepatic inflammation and lipid metabolism disorders by modulating the VDR/AMPK/SIRT1 signaling pathway, thereby improving liver health in fish models such as largemouth bass [2].

#### 2.2.4. Cell Death

Cell death can occur through various regulated pathways, including apoptotic processes such as apoptosis and necroptosis, pyroptosis, and ferroptosis [51,100,102]. Cellular demise acts as a trigger for inflammation and contributes to steatosis, leading to the onset of steatohepatitis [103].Thus, cell death represents a critical event marking the progression from benign lipid overload to inflammatory liver disease.

**Figure 6:**
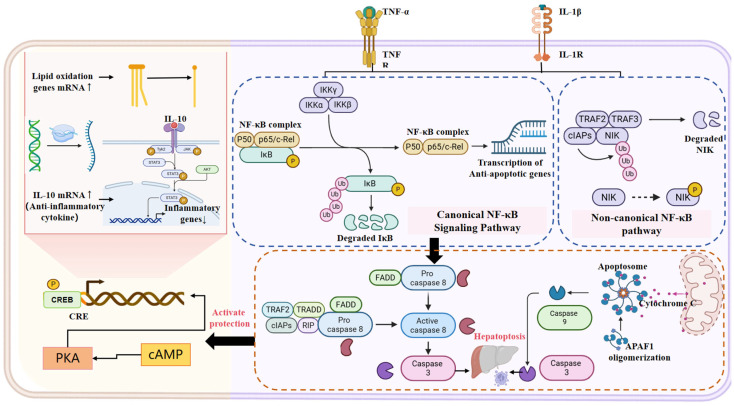
Multifunctional TNF-α Signaling Network. This diagram integrates key pathways activated by TNF-α via TNFR1. TNF-α triggers canonical NF-κB signaling (through TRADD/TRAF2/RIP1) to promote anti-apoptotic and inflammatory gene transcription, while simultaneously activating caspase-8-mediated apoptosis via FADD. In parallel, a cAMP/PKA/CREB pathway upregulates anti-inflammatory IL-10, which inhibits NF-κB via p50 homodimers. TNF-α signaling also upregulates lipid oxidation genes, linking inflammation to metabolic regulation. These intersecting pathways collectively regulate cell fate, inflammatory balance, and metabolic adaptation.

Pro-inflammatory cytokines TNF-α and IL-1β activate the p65NF-κB pathway, which plays a dual role in cell fate decisions—both activating apoptotic signals through caspase8/caspase3 and caspase9/caspase3 pathways leading to hepatoptosis, while simultaneously inhibiting overphosphorylation of NIK protein to regulate the inflammatory response (Figure 6). Apoptosis, a genetically programmed form of cell death, can be initiated by several physiological alterations. Mitochondrial dysfunction, regulated by Bcl-2 family proteins such as Bcl-2 and Bax, is a hallmark of apoptosis [104]. Mitochondrial dysfunction triggers the release of Cytochrome c, activation of the downstream effector caspase-3 (Cas-3), and ultimately leads to apoptotic changes [105]. The diagram also highlights the protective cAMP/CREB pathway, which counterbalances inflammatory processes by upregulating lipid breakdown through β-oxidation and increasing anti-inflammatory cytokine IL-10 mRNA levels, representing a crucial compensatory mechanism in liver injury (Figure 6). Accumulation of saturated fatty acids (SFAs) and free cholesterol (CH) initiates specific signaling pathways, including the JNK and mitochondrial pathways, resulting in cellular apoptosis in high-fat diet (HFD)-induced nonalcoholic fatty liver disease (NAFLD) animal models [102,106,107,108,109]. This molecular interplay between pro-apoptotic (TNF-α/NF-κB) and protective (cAMP/CREB) pathways depicted in the diagram helps explain the complex balance between cell death and survival mechanisms in fatty liver disease (Figure 6). Oxidative stress, free fatty acids (FFAs), and lipid peroxidation can also upregulate the JNK pathway in HFD-induced liver injury, promoting cellular apoptosis [109,110]. HFD induces hepatocyte apoptosis by upregulating the pro-apoptotic genes cytc and cas-3, a mechanism that has been validated in blunt snout bream. Therefore, a high-fat diet can induce cell apoptosis by activating the JNK pathway and mitochondrial pathway [111]. Apoptosis is further triggered by the upregulation of pro-apoptotic genes, such as cytochrome c (cyt c) and caspase-3 (cas-3). Consequently, a high-fat diet can promote cell apoptosis through the activation of both the JNK pathway and the mitochondrial pathway.

In comparison to apoptosis, necroptosis, and other forms of non-apoptotic cell death, ferroptosis is relatively unique due to its central involvement in iron-dependent lipid reactive oxygen species (ROS) accumulation [72,112]. Iron apoptosis, a type of programmed necrosis, precedes cell death in other types, and iron deposition triggers the onset of steatohepatitis. Ferroptosis, identified as an iron and lipid hydroperoxide-dependent non-apoptotic cell death, is related to the pathogenesis of various diseases, including neuronal dysfunction and acute renal impairment [113,114].The diagram’s emphasis on TNF-α/IL-1β-mediated apoptosis pathways complements our understanding of ferroptosis by showing alternative cell death mechanisms that may operate simultaneously or sequentially in fatty liver disease progression (Figure 5). Inhibition of iron mutations almost completely protects liver cells from necrosis and inhibits subsequent immune filtration and inflammatory reactions. Elevated levels of phosphatidylethanolamine, involved in the iron oxidation pathway in choline-deficient, ethionine-supplemented (CDE) fed mice, suggest that hepatic ferritin deposition plays a significant role in the development of steatohepatitis and could be a therapeutic target for its prevention. A potent spiroquinone derivative called livestatin-1 may inhibit iron apoptosis in cells [114].

## 3. Proposed Strategies and Solutions

### 3.1. Environmental Adaptability

The findings on cold stress-induced fatty liver underscore the importance of considering environmental adaptability when introducing fish species. Fish indigenous to the Heilongjiang River system have evolved specific mechanisms for cold tolerance, and any newly introduced varieties must also demonstrate robust cold resistance. Without a thorough assessment of temperature tolerance prior to introduction, the survival rate of the fish cannot be assured. Fish subjected to cold stress exhibit behaviors indicative of anxiety and avoidance, such as unstable swimming, abnormal posture, and aggregation behavior. When cold stress occurs, fish may cease food intake and exhibit slower swimming activities, reflecting their way of coping with stress [115,116]. Using the light–dark preference paradigm, it was observed that both cold and hot stimuli significantly enhance the avoidance of dark environments in zebrafish larvae [117]. Besides temperature adaptation, compatibility with dissolved oxygen is also crucial, making environmental factors a significant aspect of the breeding or migration process.

### 3.2. Genetic Improvement Techniques

Genetic improvement techniques have been employed in both crops and livestock to enhance food production, prevent diseases, improve product quality, and protect the environment [118]. While the application of such techniques in aquaculture is more recent compared to terrestrial agriculture, several genetically enhanced aquatic species have already demonstrated substantial economic advantages. Notable examples include the fast-growing Donaldson rainbow trout, selectively bred Norwegian Atlantic salmon, and the genetically improved farmed tilapia (GIFT) strain of Nile tilapia, all of which outperform their wild counterparts in production efficiency. The implementation of genetic modification (GM) technologies in aquaculture offers substantial potential for augmenting disease resistance, enriching omega-3 fatty acid content, and promoting ecological sustainability. Therefore, genetic modification can also hold promise in treating or preventing fatty liver disease.

Genomic selection is being actively explored in the breeding of species. In Atlantic salmon (*Salmo salar*), genomic regions associated with low lipid retention in the liver have been identified, paving the way for selective breeding programs aimed at reducing susceptibility to fatty liver disorders [119]. These advanced genetic approaches, combined with a deeper understanding of lipid metabolism networks, hold significant promise for developing next-generation aquaculture stocks with enhanced metabolic health and resilience against nutritionally induced liver pathologies.

Perhaps we can initiate research focusing on genes or proteins related to the induction of fatty liver. Homologous mutations in the ahcy gene can result in a comprehensive decrease in methylation levels, which is associated with severe hepatic steatosis in larvae, and adult ahcy heterozygotes can also exhibit hepatic steatosis [120]. Further evidence indicates that Lipin1 participates in regulating Torc1-mediated SREBP activation and lipid accumulation [60].

The antioxidant defense system in fish relies critically on enzymes such as superoxide dismutase (SOD) and catalase (CAT) to mitigate oxidative stress [39]. SOD serves as the first line of defense by neutralizing superoxide radicals and interrupting propagating chain reactions [121], while CAT detoxifies hydrogen peroxide into water and molecular oxygen, thereby safeguarding cellular integrity from peroxide-induced damage. CAT can break down hydrogen peroxide into oxygen molecules and water, thereby clearing hydrogen peroxide and protecting cells against its damage. Thus, CAT is an indispensable enzyme in biological defense [122,123].

### 3.3. Changing Feeding Habits and Contents

A growing body of evidence indicates that Nrf2 serves as a positive regulatory factor, safeguarding cells against oxidative damage [124]. Under conditions of oxidative challenge, Nrf2 activates a suite of antioxidant genes—such as HO-1, NQO1, and GST—to counteract oxidative injury and support cellular adaptation and survival [125]. The activation of Nrf2 not only inhibits adipogenesis but also enhances fatty acid β-oxidation, thereby offering protection against hepatic steatosis [126]. In HFD-induced NAFLD, other enzymes or genes exhibit inhibition of the Nrf2 pathway in the context of NAFLD [35,127,128]. Therefore, excessive fat deposits damage the Nrf2 pathway and weaken antioxidant defense.

Effective strategies to mitigate abnormal fat accumulation in fish include reducing the intake of high-fat feed and fructose. For example, studies on blunt snout bream have demonstrated that a high-fat diet promotes lipid deposition, induces pathological alterations in liver structure, disrupts the expression of lipid transport genes, and triggers endoplasmic reticulum stress [26].

Enhancing physical activity in fish is another crucial factor. Additionally, it is important to avoid overfeeding, as excessive feeding rates can significantly increase the risk of fatty liver disease. For example, overfeeding rainbow trout leads to substantial lipid buildup, resulting in liver enlargement and the proliferation of macrophages [129].

Moreover, substituting fish oil (FO) with an appropriate amount of flaxseed oil (LO) (less than 75%) can enhance lipid metabolism and antioxidant capacity. Research indicates that replacing FO with LO significantly increases serum triglyceride (TAG) levels while reducing high-density lipoprotein cholesterol (HDL). This substitution also upregulates key genes involved in lipid metabolism, such as fatty acid desaturase 6 (FAD6), which participates in the synthesis of highly unsaturated fatty acids, and acetyl-coenzyme A carboxylase (ACCo), a rate-limiting enzyme in fatty acid synthesis. Conversely, the expression of genes including SREBP-1, sterol-O-acyltransferase 2 (SOAT2, involved in cholesterol and lipoprotein absorption), and PPARα (which regulates fatty acid β-oxidation) is downregulated [130]. Notably, as dietary lipid content increases (with a concomitant reduction in fatty acid content), the levels of n-3 long-chain polyunsaturated fatty acids (LC-PUFAs), specifically eicosapentaenoic acid (EPA) and docosahexaenoic acid (DHA), decline significantly. Both DHA and EPA exhibit anti-inflammatory and lipid-lowering properties, which can alleviate high-fat diet (HFD)-induced insulin resistance (IR) by reducing hepatic steatosis and activating the PI3K/Akt signaling pathway, with DHA demonstrating more pronounced effects. Dietary supplementation with 4% DHA or EPA mitigates liver inflammation by suppressing the TLR4/NF-κB signaling pathway, with DHA showing stronger anti-inflammatory efficacy. Additionally, DHA and EPA reduce serum levels of lipopolysaccharides (LPS)—metabolic byproducts of gut microbiota—and modulate the expression of LPS receptors in the liver to varying degrees, again with DHA being more effective. These anti-inflammatory and lipid-lowering properties are further supported by a study in Nile tilapia, where DHA supplementation was shown to alleviate chemically induced liver injury by inhibiting the TLR4/NF-κB pathway, reducing inflammation and apoptosis, and improving antioxidant defenses [54]. This underscores the potential of DHA not only as a nutritional supplement but also as a therapeutic agent against fatty liver disease in aquaculture.

Furthermore, both fatty acids contribute to improved gut microbiota composition by enhancing microbial diversity and abundance, reducing pro-inflammatory bacteria (e.g., Streptococcus), and promoting beneficial bacteria (e.g., Bifidobacterium). In summary, DHA and EPA may ameliorate liver insulin resistance by regulating the gut microbiota–LPS–liver axis [131]. As an alternative to fish oil (FO), linseed oil (LO) can regulate lipid metabolism in fish. Moreover, under conditions of oxidative stress, replacing FO with LO modifies the oxidative status of fish. A lower proportion of LO supplementation is advantageous for enhancing the total antioxidant capacity of fish. However, when the LO content is excessively high, the overall antioxidant capacity of fish declines, leading to adverse effects.

Traditional Chinese Medicine (TCM) formulations typically involve combinations of herbs or natural ingredients tailored to address specific health conditions [132]. TCM formulations, natural products, and herbal compounds have shown significant efficacy in addressing various pathological aspects of MAFLD [132]. In recent years, there has been a surge in research exploring the therapeutic effects and mechanisms of TCM prescriptions, natural products, and herbal components in the treatment of MAFLD. For instance, one well-known TCM formula used for NAFLD is Dachaihu Decoction (DCHD), which comprises Chinese Thorowax Root (Chaihu, *Bupleurum falcatum* L.), Baical Skullcap Root (Huangqin, *Scutellaria baicalensis* Georgi), Rhubarb (Dahuang, *Rheum palmatum* L.), Immature Orange Fruit (Zhishi, *Citrus aurantium* L.), Pinellia Tuber (Banxia, *Pinellia ternata* (Thunb.) Makino), White Peony Root (Baishao, *Paeonia lactiflora* Pall.), Chinese Date (Dazao, *Ziziphus jujuba* Mill.), and Fresh Ginger (Shengjiang, *Zingiber officinale* Roscoe) [10]. Similarly, glycyrrhizin (GL), a triterpene glycoside commonly used as a food sweetener or active pharmaceutical ingredient, exhibits diverse medicinal properties. Research suggests that GL has therapeutic potential for MAFLD due to its ability to inhibit NLRP3 inflammasome activation, reduce adipose tissue inflammation, improve insulin sensitivity, and alleviate hepatic inflammation and fibrosis in animal models [132]. Another effective TCM prescription for NAFLD is Yu Hua Tan Tong Luo (QYHTTL), which includes Bupleuri Radix, Scutellariae Radix, Pinelliae Rhizoma, Codonopsis Radix, Glycyrrhizae Radix et Rhizoma, Jujubae Fructus, RJ, Morindae Officinalis Radix, and Oldenlandia Diffusa. Studies indicate that QYHTTL significantly reduces hepatic aminotransferase levels and improves lipid profiles [133] in combination with conventional therapy, has been shown to significantly improve outcomes in non-alcoholic fatty liver disease, representing a safe and effective treatment option for NAFLD [134]. Overall, these findings highlight the potential of TCM prescriptions and natural compounds in managing MAFLD, offering promising avenues for further research and clinical application. Vitamin E plays a pivotal role in energy metabolism. First discovered in 1922, α-tocopherol (Vitamin E) was initially recognized for its ability to prevent embryonic death. A deficiency in Vitamin E can trigger the peroxidation of docosahexaenoic acid (DHA), depleting phospholipids that contain DHA, particularly phosphatidylcholine, and subsequently leading to choline depletion. This heightened lipid peroxidation further accelerates NADPH oxidation, redirecting glucose toward the pentose phosphate pathway. Vitamin E deficiency is also linked to mitochondrial dysfunction, which disrupts energy homeostasis. As an integral component of the antioxidant network, Vitamin E demonstrates significant antioxidant properties [135]. These mechanisms present promising research targets for addressing fatty liver disease. Studies have shown that Vitamin E deficiency impairs fat metabolism in tilapia fry, resulting in increased fat accumulation, while Vitamin E supplementation can effectively counteract these adverse effects [136].

A subsequent study by the same team provided deeper insights into the dose-dependent efficacy of tea polyphenols (TPs). In GIFT tilapia fed a high-fat diet, supplementation with 50 mg/kg TPs was more effective than 200 mg/kg in reducing hepatic lipid deposition and improving survival. The lower dose enhanced immunity and antioxidant capacity, likely by promoting energy supply through increased fatty acid β-oxidation (upregulated CPT1α). In contrast, the higher dose primarily inhibited lipid absorption and showed weaker beneficial effects, highlighting the importance of optimal dosing in nutritional interventions [137].

The application of probiotics, prebiotics, and synbiotics has emerged as a promising strategy for managing non-alcoholic fatty liver disease (NAFLD). Evidence indicates that these gut microbiota-targeted interventions can reverse NAFLD-associated dysbiosis, leading to improvements in disease-related biomarkers. This approach has been shown to reduce liver damage, inflammation, and insulin resistance linked to NAFLD [138]. In both human and animal studies focused on NAFLD and non-alcoholic steatohepatitis (NASH), the administration of probiotics, prebiotics, and synbiotics has resulted in significant reductions in serum liver aminotransferases, inflammatory cytokines, and chemokines. Emerging evidence highlights the critical role of the gut–liver axis in the pathogenesis of fatty liver disease. Dysbiosis of the gut microbiota can disrupt intestinal barrier integrity, leading to increased translocation of bacterial endotoxins (e.g., lipopolysaccharides, LPS) into the portal circulation, which triggers hepatic inflammation and metabolic dysfunction [53]. Recent studies in fish have shown that high-fat diets alter the composition of gut microbiota, reducing beneficial bacteria (e.g., Lactobacillus, Bifidobacterium) while increasing pro-inflammatory taxa, thereby exacerbating hepatic steatosis [32]. Modulation of the gut microbiome through probiotics, prebiotics, or synbiotics represents a promising strategy to mitigate fatty liver progression [139]. Exploring these solutions may help us find the door to further increasing the efficiency of aquaculture.

Sodium citrate has emerged as a potential metabolic modulator in aquafeeds. While it demonstrates positive effects in promoting the deposition of protein and lipids, its capacity to induce hyperglycemia, insulin resistance, and underlying inflammatory responses warrants careful consideration [20]. Therefore, its practical application must be carefully evaluated, taking into account fish species, basal diet composition, and dosage to mitigate potential adverse metabolic side effects.

Excessive carbohydrate intake in fish can lead to hepatic steatosis, impairing normal liver function. Adding a specific amount of choline to feed can prevent hepatic steatosis even in high-carbohydrate diets. Although high-carbohydrate feed isn’t nutritionally balanced, it should still be utilized when protein is scarce. Choline serves as a vital methyl donor and plays a crucial role in fat metabolism [140]. Vitamins that serve as methyl donors and are essential for phospholipid synthesis, such as folic acid and vitamin B12, when deficient in feed, can directly disrupt fat transport and contribute to the development of fatty liver disease [9].

Furthermore, the latest research has uncovered the pivotal role of nutritional immunology in liver health. Vitamin D3 (VD3), a vital fat-soluble vitamin, demonstrates functions that extend far beyond traditional calcium and phosphorus metabolism regulation. Recent studies on perch Largemouth bass revealed that dietary supplementation with VD3 (1822 IU/kg) significantly enhances the liver’s ability to resist infections by Nocardia seriolae through activation of vitamin D receptors (VDRs) [141].

### 3.4. Rapamycin

In recent studies, researchers have demonstrated that mTOR (the mechanistic target of rapamycin) represents a key potential pathway in the development of NASH. As a conserved nutrient-sensing pathway, mTOR integrates signals from growth factors, energy status, and amino acids to regulate anabolic and catabolic processes [142]. Furthermore, rapamycin, an inhibitor of mTOR signaling, has been shown to reverse NASH in all of our experimental models. One study found that supplementing feed with 30 mg/kg of rapamycin reduced growth performance and feed utilization efficiency in juvenile turbot. It also inhibited the TOR signaling pathway and disrupted glycolysis and lipid synthesis processes [61]. This growth-suppressing effect poses a significant challenge for its application in aquaculture, where maximizing biomass production is often a primary goal.

In addition to its impact on lipid production and accumulation, rapamycin plays a significant role in reversing other processes. Among these, Torc1 primarily mediates lipid accumulation in the liver caused by fructose or oxidative stress by regulating fat production. Mechanistically, this occurs through the phosphorylation and inhibition of Lipin 1, which in turn promotes the nuclear localization and transcriptional activity of SREBP-1, a master regulator of lipogenesis [60]. In contrast, treatment with ER stress-inducing agents did not significantly increase lipogenic gene expression but led to a decline in autophagy-related genes. Rapamycin may counteract this decline. Additionally, rapamycin appears to alleviate fructose- and valinomycin-induced steatosis by modulating the expression of lipogenic genes and transcription factors.

The utilization of rapamycin in agriculture remains limited. Its application in fish farming and other aquaculture practices is still largely in the exploratory phase and has not yet developed into a mature technological system. Furthermore, the mechanism of action of rapamycin is complex, and the long-term effects and potential risks associated with its use in agricultural production are not fully understood. These risks may include immunosuppression under certain conditions, as the mTOR pathway is also crucial for immune cell activation and function [143].

Given these complexities, targeting more direct components of the lipid metabolic machinery may offer a safer alternative. For example, L-carnitine directly facilitates mitochondrial β-oxidation without the pleiotropic effects associated with mTOR inhibition. Its efficacy in reducing hepatic lipid deposition and improving systemic metabolic health in HFD-fed fish highlights the potential of such targeted nutrient-based interventions [51].

## 4. Conclusions and Future Prospects

Fatty liver disease, a major concern in aquaculture, causes metabolic dysfunction, impaired immunity, and higher mortality rates among farmed fish. This condition poses substantial economic and sustainability challenges for the aquaculture industry. The rising incidence of fatty liver is closely linked to modern intensive farming practices, including high-fat diets, environmental stressors, and overfeeding. Understanding the mechanisms and solutions for fatty liver is not only critical for improving fish health and aquaculture productivity but also offers valuable insights into similar metabolic diseases in humans, such as non-alcoholic fatty liver disease (NAFLD).

Feed composition, particularly high-fat diets and fructose, plays a central role in inducing fatty liver by promoting lipid accumulation, oxidative stress, and inflammation. Environmental stressors, such as cold stress and hypoxia, exacerbate metabolic dysfunction and liver damage. Key molecular pathways, including mTOR, PPAR, and Nrf2, are implicated in lipid metabolism and oxidative stress responses.

Reducing high-fat feed, incorporating omega-3 fatty acids (e.g., DHA and EPA), and using Traditional Chinese Medicine (TCM) prescriptions show promise in mitigating fatty liver. Optimizing water quality and temperature can reduce stress-induced metabolic disruptions. Techniques like genetic modification and the use of rapamycin (an mTOR inhibitor) offer novel approaches to controlling lipid metabolism and inflammation.

Subsequent investigations should aim to unravel the regulatory mechanisms governing lipid metabolism. Concurrently, evaluating the potential of probiotic, prebiotic, and synbiotic interventions to restore gut–liver axis homeostasis is essential, thereby guiding the development of sustainable therapeutic strategies. Leveraging findings from fish models to advance research on human NAFLD and metabolic syndromes. Developing standardized feeding protocols and environmental guidelines to minimize fatty liver risks in aquaculture.

In summary, fatty liver in fish has a dual nature. It is a vital physiological process for energy storage and reproduction, but it becomes a harmful metabolic disorder when balance is lost. In aquaculture, the high rate of fatty liver disease is a maladaptation, where intensive farming conditions pathologically disrupt this normally beneficial process.

## Data Availability

No new data were created or analyzed in this study. Data sharing is not applicable to this article.
